# Neonatal Intensive Care Unit Admissions among Preterm Babies in a Tertiary Care Centre: A Descriptive Cross-sectional Study

**DOI:** 10.31729/jnma.7240

**Published:** 2022-04-30

**Authors:** Sahisnuta Basnet, Suraj Adhikari, Jyoti Jha, Mahendra Raj Pandey

**Affiliations:** 1 Department of Pediatrics, Manipal College of Medical Sciences, Phulbari, Pokhara, Nepal; 2 Manipal College of Medical Sciences, Phulbari, Pokhara, Nepal; 3 Department of Obstetrics and Gynecology, Manipal College of Medical Sciences, Phulbari, Pokhara, Nepal

**Keywords:** *morbidity*, *mortality*, *neonatal intensive care unit*, *preterm infants*

## Abstract

**Introduction:**

Preterm babies are born before 37 completed weeks of gestation. It is an important cause of neonatal morbidity and mortality. This study aimed to find out the prevalence of neonatal intensive care unit admissions among preterm babies in a tertiary care centre.

**Methods:**

A descriptive cross-sectional study on a total of 133 preterm infants was conducted in a tertiary care centre from November, 2020 to April, 2021 with ethical approval from the Institutional Review Committee (Reference number: 380). Preterm babies who met the eligibility criteria were included in the study. Convenience sampling was done. Data were analysed using the Statistical Package for the Social Sciences version 20.0. Point estimate at 95% Confidence Interval was calculated along with frequency and percentage for binary data.

**Results:**

Out of 133 preterm babies, 54 (40.60%) (32.25-48.95 at 95% Confidence Interval) had neonatal intensive care unit admissions. Hyaline membrane disease was the most common illness in preterm neonates 34 (62.96%) followed by neonatal sepsis 20 (37.03%).

**Conclusions:**

The prevalence of neonatal intensive care unit admissions among preterm babies in our study was similar to other studies done in similar settings. Preterm newborns are significantly vulnerable and maternal risk factors should be taken into account. Anticipated preterm deliveries should have mandatory institutional delivery and adequate postnatal care is needed to improve the outcomes of preterm babies.

## INTRODUCTION

Prematurity is the leading cause of death in children under 5 years and 60% of these births occur in Africa and South Asia.^1^ In Nepal, the neonatal mortality rate is 20 deaths per 1000 live births, and 33% of these deaths were attributed to preterm birth complications.^2^ Complications arising from preterm births are the single largest direct cause of neonatal deaths.^3^ Common causes of preterm births are maternal infections, diabetes, hypertension, smoking, older age, primiparily, foetal distress, multiple gestations, antepartum haemorrhage, and poor access to antenatal care services during pregnancy.^4^

In order to achieve the Sustainable Development Goal 3 target of reducing the neonatal mortality rate to 12/1000 live births by 2030, it is vital to critically analyse the factors leading to preterm morbidity and mortality.^5^

This study aims to find out the prevalence of neonatal intensive care unit admissions among preterm babies in a tertiary care centre.

## METHODS

A descriptive cross-sectional study was conducted in the Neonatal Intensive Care Unit (NICU) of Manipal Teaching Hospital, Pokhara, Nepal from November, 2020 to April, 2021. Ethical approval was obtained from the institutional review committee (Reference number: 380). All preterm (those neonates born before 37 completed weeks of gestation) admitted to the NICU of the hospital were included in the study. Neonates whose guardians did not give consent for the study were excluded. A convenience sampling was done. The sample size was calculated using the following formula:

n = (Z^2^ × p × q) / e^2^

  = (1.96^2^ × 0.5 × 0.5) / 0.09^2^

  = 119

Where,

n = minimum required sample size
Z = 1.96 at 95% Confidence Interval (CI)
p = prevalence taken as 50% for maximum sample size calculation
q = 1-p
e = margin of error, 9%

The calculated minimum required sample size was 119. However, we included 133 subjects in our study. Data including maternal and neonatal demographics, maternal risk factors, the status of the neonate during admission and daily parameters of the neonate was collected based on a structured proforma. Detailed assessment of the cause of mortality and morbidity was assessed and maternal records were accessed whenever necessary.

The gestational age of neonates was calculated based on the Last Menstrual Period (LMP). If LMP was unknown or the menstrual pattern was irregular, Modified New Ballard Score was used to calculate gestational age by trained personnel. Weight was measured by using a digital electronic weighing machine with a resolution of ±5 grams and length was measured with an infant metre to an accuracy of 0.1 cm. Data analysis was done using the Statistical Package for the Social Sciences version 20.0. Categorical data was analysed and presented in range or mode and numerical data was presented in mean and standard deviation. Point estimate at 95% Confidence Interval was calculated along with frequency and percentage for binary data.

## RESULTS

Out of 133 preterm babies, 54 (40.60%) (32.25-48.95 at 95% Confidence Interval) had neonatal intensive care unit admissions. There were 34 (62.96%) males and 20 (37.04%) females with a male:female ratio of 1.7:1. The maternal and neonatal characteristics are presented (Table 1).

**Table 1:** Maternal and neonatal demographics of NICU admitted preterm cases (n= 54).

Parameters		n (%)
Age of the mother in years	<35	49 (90.74)
	>35	5 (9.26)
	Mean±SD	25.65± 5.61
Age of neonate at admission	<24 hours	37 (68.52)
	>24 hours	17 (31.48)
	Mean±SD	2.65±3.43
Gender of neonate	Male	34 (62.96)
	Female	20 (37.04)
**Birth weight in grams (Mean±SD)**		1657±393.14
Parity	1	30 (55.56)
	2	19 (35.19)
	3	4 (7.41)
	4	1 (1.85)
Education of mother	Illiterate	1 (1.85)
	Basic	11 (20.37)
	Secondary	26 (48.15)
	College	16 (29.63)
Residence	Rural	22 (40.74)
	Urban	32 (59.26)
Ethnicity	Brahmin	18 (33.33)
	Chhetri	6 (11.11)
	Janajati	20 (37.03)
	Dalit	10 (18.52)
Occupation of mother	Housewife	33 (61.11)
	Business	10 (18.52)
	Service	11 (20.37)
Number of ANC visits	<4	17 (31.48)
	>4	37 (68.52)
**Monthly family income in rupees**		25,000
Median (IQR)		(20,000-50,000)
Number of babies	Singleton	52 (96.30)
	Twins	2 (3.70)
Types of delivery	Vaginal	19 (35.19)
	Caesarean section	35 (64.81)
Indication of Cesarean section	Impending eclampsia	12 (34.29)
	Preterm labour	7 (20.00)
	Severe oligohydramnios	5 (14.29)
	Foetal distress	5 (14.29)
	Previous caesarean section	2 (5.71)
	Twins	2 (5.71)
	Non-progression of labour	1 (2.86)
	Abruptio placenta	1 (2.86)
Inborn		41 (75.93)
Outborn		13 (24.07)
**Duration of hospital stay in days, Mean±SD**		8.13±4.84
Final outcome	Discharged	36 (66.67)
	Leave Against Medical Advice (LAMA)	7 (12.96)
	Death	11 (20.37)

The most common cause of preterm birth was pregnancy-induced hypertension 17 (31.48%) followed by premature rupture of membranes 16 (29.63%). The details of maternal risk factors are presented (Table 2).

**Table 2:** Maternal risk factors among NICU admitted preterm babies (n= 54).

Maternal risk factors	n (%)
None	10 (18.52)
Pregnancy Induced Hypertension (PIH)	17 (31.48)
Premature Rupture of Membrane (PROM)	16 (29.63)
Urinary Tract Infection (UTI)	4 (7.41)
Anaemia	3 (5.56)
Previous preterm delivery	2 (3.70)
Previous caesarean section	1 (1.85)
Hypothyroidism	1 (1.85)
Duration of PROM in hours (Mean±SD)	4.20±7.65

Hyaline Membrane Disease (HMD) was the most common illness in preterm neonates 34 (62.96%) followed by neonatal sepsis 20 (37.03%). The associated illness in admitted preterm neonates (Table 3).

**Table 3:** Illness in neonates during admission (n= 54).

Associated illness in neonates	n (%)
Hyaline membrane disease	34 (62.96)
Sepsis	20 (37.03)
Neonatal hyperbilirubinemia	3 (5.56)
Necrotizing enterocolitis	3 (5.56)
Perinatal asphyxia	1 (1.85)
Meconium aspiration syndrome	1 (1.85)
Intrauterine growth retardation	1 (1.85)

The overall mortality was 11(20.37%). The finaloutcomes according to the diagnosis are presented (Table 4).

**Table 4:** Problems during admission and the final outcome (n= 54).

Illness in neonates	Discharged n (%)	LAMA n (%)	Death n (%)
Hyaline membrane disease	19 (55.89)	6 (17.65)	9 (26.47)
Sepsis	17 (85.00)	1 (5.00)	2 (10.00)
Necrotizing enterocolitis	-	1 (33.33)	2 (66.67)
Neonatal hyperbilirubinemia	3 (100)	-	-
Meconium aspiration syndrome	1 (100)	-	-
Perinatal asphyxia	1 (100)	-	-
Intrauterine growth retardation	1 (100)	-	-

## DISCUSSION

Rates of preterm deliveries are seen to be on the rise in spite of concentrated research into its epidemiology, causes and management. Regardless of the cause, preterm birth imposes substantial financial, and emotional burdens and suffering on the infant, family, health care system and society. The present study highlights preterm morbidity and mortality in a tertiary healthcare centre in western Nepal. The relatively higher incidence rate in our study could be attributed to a lack of awareness about institutional delivery and poor antenatal visits, as is prevalent in low-income countries like Nepal. Out of the 133 preterm babies in our institute, 54 (40.60%) required NICU admissions.

Male predominance was seen in our study with the male:female ratio of 1.7:1. This finding was consistent with the finding of other studies which had shown a male predominance.^6,7^ This trend is common in South Asian regions where male preference is prevalent and males are given more care. The present study showed that 49 (90.74%) of preterms were born to mothers less than 35 years as compared to 5 (9.26%) preterms born to mothers with ages more than 35 years. Caesarean section was the mode of delivery in 35 (64.81%) cases in our study which is higher than those reported by other studies.^8,9^ This high number can be explained by the fact that our institute is a tertiary care centre that receives high-risk pregnancies with co-morbidities, requiring caesarean section.

The most common maternal cause associated with preterm birth in our study was pregnancy-induced hypertension 17 (31.48%) followed by premature rupture of membranes 16 (29.63%). The most common pregnancy complication associated with low birth weight was hypertensive disorders of pregnancy (17.49%) in another study.^10^ However, maternal anaemia, multiple births and malformations were attributed as common causes of preterm deliveries in several other studies.^11-13^

Seven comorbidities were identified in the preterms enrolled in this study and hyaline membrane disease (HMD) was the most common illness 34 (62.96%). One of the studies had a similar finding in their study showing HMD as a major cause of morbidity among preterm babies.^6^ Some studies. have however shown jaundice as the most common morbidity in preterm babies.^14,15^ These findings are not surprising as both HMD and hyperbilirubinemia are very common because of developmental immaturity of the lungs and liver in the preterm population. Sepsis was shown to be the major cause of co-morbidity in several studies.^6,16^ The relatively lower incidence of 20 (37.03%) in our study may be accounted for by the fact that the majority of the preterms in our study were inborn.

The overall mortality was 11 (20.37%) with the highest number of deaths among babies with hyaline membrane disease 9 (26.47%) followed by sepsis 2 (10.00%) and necrotizing enterocolitis 2 (66.67%). A similar trend was seen in studies where the major causes of preterm mortality were also attributed to hyaline membrane disease.^7,10^ However, the mortality rate in a study was much lower than in our study.^7^

The limitation of our study was that this was a single hospital-based study so it may not reflect the patterns seen in all hospitals. Also, the outcomes of babies who left against medical advice or those who were referred could not be reflected in this study. Finally, errors may have occurred in the calculation of gestational age where respondents were unable to recall their last menstrual period. In these cases, the New Ballard Score was used and this itself is prone to reduced accuracy when performed after 48 hours of life and this may have occurred in some of the preterms who were not admitted immediately after birth.

## CONCLUSIONS

The prevalence of NICU morbidities in our hospital was similar to the findings in similar settings. Though advances have been made in terms of neonatal care, preterm neonates are a significant vulnerable population involving both morbidity and mortality. Commonly seen complications such as HMD and sepsis results in higher mortalities. Deliveries that are anticipated to be preterm should have mandatory deliveries in institutions where high-level NICU care is available which may then improve preterm outcomes.
